# Domiciliary VR-Based Therapy for Functional Recovery and Cortical Reorganization: Randomized Controlled Trial in Participants at the Chronic Stage Post Stroke

**DOI:** 10.2196/games.6773

**Published:** 2017-08-07

**Authors:** Belén Rubio Ballester, Jens Nirme, Irene Camacho, Esther Duarte, Susana Rodríguez, Ampar Cuxart, Armin Duff, Paul F.M.J Verschure

**Affiliations:** ^1^ Laboratory of Synthetic Perceptive, Emotive and Cognitive Systems, Center of Autonomous Systems and Neurorobotics Department of Information and Communication Technologies Pompeu Fabra Barcelona Spain; ^2^ Servei de Medicina Física I Rehabilitació Institut Hospital del Mar d’Investigacions Mèdiques Hospitals del Mar I l’Esperança Barcelona Spain; ^3^ Servei de Medicina Física i Rehabilitació Hospital Universitari Vall dHebron Barcelona Spain; ^4^ ICREA Institució Catalana de Recerca i Estudis Avançats Barcelona Spain; ^5^ Institute for Bioengineering of Catalonia (IBEC) The Barcelona Institute of Science and Technology Baldiri Reixac 10-12, 08028 Barcelona Spain

**Keywords:** stroke, movement disorder, recovery of function, neuroplasticity, transcranial magnetic stimulation, physical therapy, hemiparesis, computer applications software

## Abstract

**Background:**

Most stroke survivors continue to experience motor impairments even after hospital discharge. Virtual reality-based techniques have shown potential for rehabilitative training of these motor impairments. Here we assess the impact of at-home VR-based motor training on functional motor recovery, corticospinal excitability and cortical reorganization.

**Objective:**

The aim of this study was to identify the effects of home-based VR-based motor rehabilitation on (1) cortical reorganization, (2) corticospinal tract, and (3) functional recovery after stroke in comparison to home-based occupational therapy.

**Methods:**

We conducted a parallel-group, controlled trial to compare the effectiveness of domiciliary VR-based therapy with occupational therapy in inducing motor recovery of the upper extremities. A total of 35 participants with chronic stroke underwent 3 weeks of home-based treatment. A group of subjects was trained using a VR-based system for motor rehabilitation, while the control group followed a conventional therapy. Motor function was evaluated at baseline, after the intervention, and at 12-weeks follow-up. In a subgroup of subjects, we used Navigated Brain Stimulation (NBS) procedures to measure the effect of the interventions on corticospinal excitability and cortical reorganization.

**Results:**

Results from the system’s recordings and clinical evaluation showed significantly greater functional recovery for the experimental group when compared with the control group (1.53, SD 2.4 in Chedoke Arm and Hand Activity Inventory). However, functional improvements did not reach clinical significance. After the therapy, physiological measures obtained from a subgroup of subjects revealed an increased corticospinal excitability for distal muscles driven by the pathological hemisphere, that is, abductor pollicis brevis. We also observed a displacement of the centroid of the cortical map for each tested muscle in the damaged hemisphere, which strongly correlated with improvements in clinical scales.

**Conclusions:**

These findings suggest that, in chronic stages, remote delivery of customized VR-based motor training promotes functional gains that are accompanied by neuroplastic changes.

**Trial Registration:**

International Standard Randomized Controlled Trial Number NCT02699398 (Archived by ClinicalTrials.gov at https://clinicaltrials.gov/ct2/show/NCT02699398?term=NCT02699398&rank=1)

## Introduction

After initial hospitalization, many stroke patients return home relatively soon despite still suffering from impairments that require continuous rehabilitation [1]. Therefore, ¼ to ¾ of patients display persistent functional limitations for a period of 3 to 6 months after stroke [2]. Although clinicians may prescribe a home exercise regimen, reports indicate that only one-third of patients actually accomplish it [3]. Consequently, substantial gains in health-related quality of life during inpatient stroke rehabilitation may be followed by equally substantial declines in the 6 months after discharge [4]. Multiple studies have shown, however, that supported discharge combined with at home rehabilitation services does not compromise clinical inpatient outcomes [5-7] and may enhance recovery in subacute stroke patients [8]. Hence, it is essential that new approaches are deployed that help to manage chronic conditions associated with stroke, including domiciliary interventions [9] and the augmentation of current rehabilitation approaches in order to enhance their efficiency. There should be increased provision of home-based rehabilitation services for community-based adults following stroke, taking cost-effectiveness, and a quick family and social reintegration into account [10].

One of the latest approaches in rehabilitation science is based on the use of robotics and virtual reality (VR), which allow remote delivery of customized treatment by combining dedicated interface devices with automatized training scenarios [10-12]. Several studies have tested the acceptability of VR-based setups as an intervention and evaluation tool for rehabilitation [13-15]. One example of this technology is the, so called, Rehabilitation Gaming System (RGS) [16], which has been shown to be effective in the rehabilitation of the upper extremities in the acute and the chronic phases of stroke [13]. However, so far little work exists on the quantitative assessment of the clinical impact of VR based approaches and their effects on neural reorganization that can directly inform the design of these systems and their application in the domiciliary context. The main objective of this paper is to further explore the potential and limitations of VR technologies in domiciliary settings. Specifically, we examine the efficacy of a VR-based therapy when used at home for (1) assessing functional improvement, (2) facilitating functional recovery of the upper-limbs, and (3) inducing cortical reorganization. This is the first study testing the effects of VR-based therapy on cortical reorganization and corticospinal integrity using NBS.

## Methods

### Design

We conducted a parallel-group, controlled trial in order to compare the effectiveness of domiciliary VR-based therapy versus domiciliary occupational therapy (OT) in inducing functional recovery and cortical reorganization in chronic stroke patients.

### Participants

Participants were first approached by an occupational therapist from the rehabilitation units of Hospital Esperanza and Hospital Vall d’Hebron from Barcelona to determine their interest in participating in a research project. Recruited participants met the following inclusion criteria: (1) mild-to-moderate upper-limbs hemiparesis (Proximal MRC>2) secondary to a first-ever stroke (>12 months post-stroke), (2) age between 45 and 85 years old, (3) absence of any major cognitive impairment (Mini-Mental State Evaluation, MMSE>22), and (4) previous experience with RGS in the clinic. The ethics committee of clinical research of the Parc de Salut Mar and Vall d’Hebron Research Institute approved the experimental guidelines. Thirty-nine participants at the chronic stage post-stroke were recruited for the study by two occupational therapists, between October 2011 and January 2012, and were assigned to a RGS (n=20) or a control group (n=19) using stratified permuted block randomization methods for balancing the participants’ demographics and clinical scores at baseline (Table 1). One participant in the RGS group refused to participate. Prior to the experiment, participants signed informed consent forms. This trial was not registered at or before the onset of participants’ enrollment because it is a pilot study that evaluates the feasibility of a prototype device. However, this study was registered retrospectively in ClinicalTrials.gov and has the identifier NCT02699398.

### Instrumentation

#### Description of the Rehabilitation Gaming System

**Figure 1:**
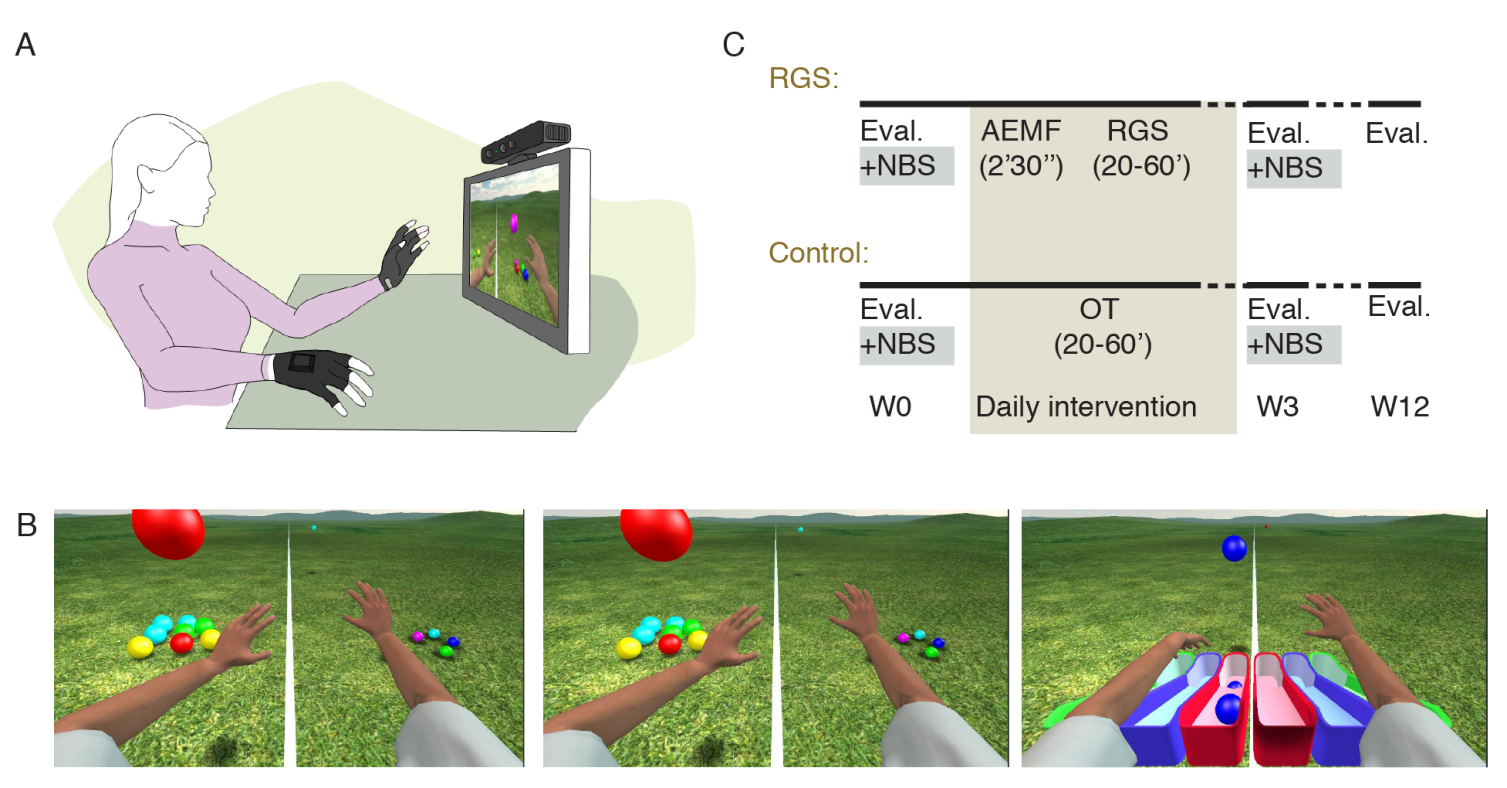
Experimental setup and protocol: (A) Movements of the user’s upper limbs are captured and mapped onto an avatar displayed on a screen in first person perspective so that the user sees the movements of the virtual upper extremities. A pair of data gloves equipped with bend sensors captures finger flexion. (B) The Spheroids is divided into three subtasks: hit, grasp, and place. A white separator line divides the workspace in a paretic and non-paretic zone only allowing for ipsilateral movements.(C) The experimental protocol. Evaluation periods (Eval.) indicate clinical evaluations using standard clinical scales and Navigated Brain Stimulation procedures (NBS). These evaluations took place before the first session (W0), after the last session of the treatment (day 15, W3), and at follow-up (week 12, W12).

The RGS integrates a paradigm of goal-directed action execution and motor imagery [17], allowing the user to control a virtual body (avatar) through an image capture device (Figure 1). For this study, we developed training and evaluation scenarios within the RGS framework. In the Spheroids training scenario (Figure 1), the user has to perform bilateral reaching movements to intercept and grasp a maximum number of spheres moving towards him [16]. RGS captures only joint flexion and extension and filters out the participant’s trunk movements, therefore preventing the execution of compensatory body movements [18]. This task was defined by three difficulty parameters, each of them associated with a specific performance descriptor: (1) different trajectories of the spheres require different ranges of joint motion for elbow and shoulder, (2) the size of the spheres require different hand and grasp precision and perceptual abilities, and (3) the velocity of the spheres require different movement speeds and timing. All these parameters, also including the range of finger flexion and extension required to grasp and release spheroids, were dynamically modulated by the RGS Adaptive Difficulty Controller [19] to maintain the performance ratio (ie, successful trials over the total trials) above 0.6 and below 0.8, optimizing effort and reinforcement during training [20].

#### Description of the Evaluation Scenario

Designing automated evaluation tools to be used at-home in a non-supervised setup could provide objective and frequent measurements of recovery, offering valuable information to clinicians and primary users, and driving autonomous rehabilitation technologies. We, therefore, developed the Automated Evaluation of Motor Function (AEMF), a VR-based evaluation scenario for the assessment of upper-limb motor function that was designed to operate under non-supervised conditions.

#### Description of the Automated Evaluation of Motor Function (AEMF)

In order to assess proximal and distal motor function, the AEMF scenario is divided into two separated tasks. In task 1, participants were asked to perform planar wiping movements with their arms to clear a virtual surface covered with small cubes. In task 2, participants were instructed to squeeze a virtual object by flexing and extending their fingers. In order to guarantee that the AEMF tasks were correctly understood, each of these was first performed using the non-paretic limb and then with the paretic limb. Participants did not receive any explicit feedback (ie, knowledge of results) about their overall performance. During task execution, we collected data of hand position and joint rotation (fingers, elbows, and shoulders) to compute three main performance descriptors: the horizontal planar area covered, finger flexion, and extension.

### Experimental Protocol

In order to test the effectiveness of VR in the domiciliary context, each participant received daily home-based upper-limb rehabilitation during 5 weekly days, for 3 consecutive weeks. The RGS group followed a home-based training paradigm based on the Spheroids scenario (Figure 1), comprising 3 consecutive subtasks: Hit, Grasp, and Place, with a total duration of 20 minutes, 6 minutes, and 40 seconds each. Participants in the RGS group completed the Automated Evaluation of Motor Function once a day, before the training session, which lasted 2 minutes and 30 seconds. We delivered the system and trained the participants and their corresponding caregivers to use the system without supervision. The control group performed a 20 minutes OT task at home, without assistance, which consisted of horizontal and vertical stacking and unstacking of plastic cups with their right and left hand consecutively. This task was designed by an occupational therapist to match the movements trained during the RGS task. At the end of the therapy, the participants reported to have completed a minimum of 1 session a day. In the RGS group, the therapy time was similarly split between 10 minutes of activity with the affected hand and 10 minutes with the less affected hand. All participants were asked to perform a minimum of 1 and a maximum of 3 training sessions a day.

### Outcome Measures

All participants’ motor function was evaluated at day 1, day 15 of the rehabilitation program, and week 12 follow-up (Figure 1), using 8 standard clinical scales. Evaluations were carried out by two occupational therapists who were not blinded to treatment assignment. Primary outcomes were the improvement in the upper extremity section of the Fugl-Meyer Assessment (UE-FM) [21], and the Chedoke Arm and Hand Activity Inventory (CAHAI) [22]. Secondary outcomes were improvements in Barthel Index (BI) [23], Ashworth Scale for distal (ASd) and proximal upper limb (ASp) [24], Medical Research Council Scale for distal (MRCd) and proximal upper limb (MRCp) [25], and grip force. In addition, we used the Hamilton Scale to assess mood disorders [26], and the Visual Analog Scale (VAS) to evaluate shoulder pain [27].

Both during the training and evaluation sessions, we captured the user’s movements and mapped them onto a biomechanical model of the upper limbs. Specifically, virtual movements were controlled by the angles of the users’ joints measured by a motion capture device at 30Hz (Kinect, Microsoft, USA). The range of finger flexion was captured by a pair of data gloves (DGTech Engineering Solutions, Bazzano, Italy) equipped with bend sensors, measures range from 0 to 1, indicating maximum extension and maximum flexion respectively.

**Figure 2:**
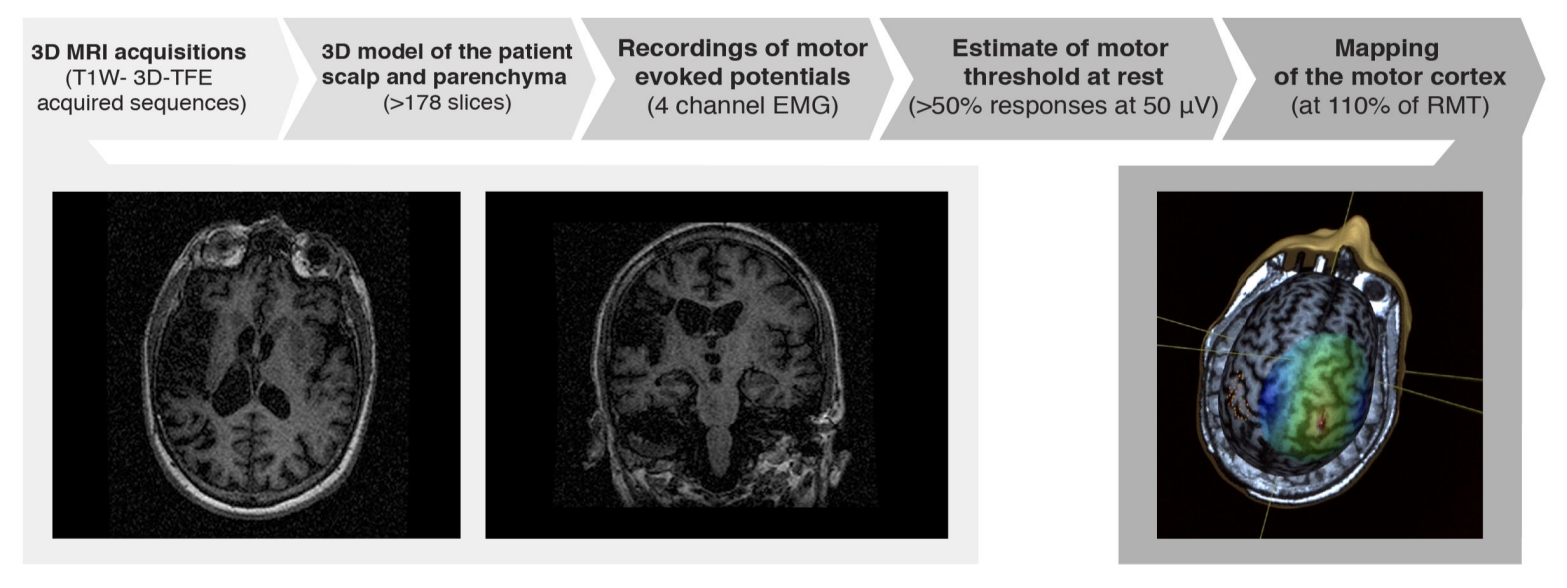
Navigated Brain Stimulation (NBS) procedure. Bottom right: axial and coronal view of a magnetic resonance imaging (MRI) scan at the level of the stroke for one of the participants in the experimental group showing a partial anterior circulation infarct due to an embolism. Bottom right: Example of NBS mapped cortical motor representations; colored areas indicate the targeted cortical sites.

Navigated Brain Stimulation (NBS) procedures [28] were used to assess training-induced changes in the functional integrity of the pyramidal tract and cortical maps in the primary motor area (M1). A total of 17 participants (3 of them assigned to the control group) accepted to participate in the NBS procedure, which was conducted for each subject before and after treatment (Figure 2). A 3-Tesla magnet (Philips Achieva) was used for 3D MRI acquisitions. In order to faithfully build a 3D model of the participant’s scalp and parenchyma we used T1W- 3D-TFE acquired sequences comprising a minimum of 178 slices. For nTMS mapping we used a butterfly coil (MC-B70, Medtronic, Alpine, USA), and magnetic stimulation equipment (Mag Pro-30 with MagOption, Medtronic, Alpine, USA) synchronized with a three-dimensional tracking system (Navigated Brain System, Nexstim, Eximia, Finland). Motor evoked potentials (MEPs) were recorded using surface electrodes (Ambu, Neuroline 700), connected to a 4 channel electromyographic (EMG) system (Key-Point net, Medtronic, USA). Data collected during NBS was analyzed to estimate the motor threshold at rest (RMT) for abductor pollicis brevis (APB) and extensor-carpi radialis (ECR) for each participant. The RMT was defined as the intensity of TMS stimulus needed to obtain more than 50% of responses with amplitudes over 50 μV. After finding the RMT of both muscles we proceeded to draw the cortical maps of both the healthy and pathological hemisphere in each participant. Maps were drawn at 110% of RMT, a percentage commonly used to avoid no-response spots and suppressive effects. When no-response was found on the pathological side we incremented the stimulus intensity stepwise in a logarithmic fashion (ie, 110%, 120%, and 140%) until the maximum stimulator output was reached. To determine the boundaries, we stopped searching a particular direction until two no-response points aligned in the same vector and direction or when the sulcus boundaries were reached. After processing the data, we characterized cortical representations of APB and ECR and corticospinal connectivity in each cerebral hemisphere by estimating the centroid of the cortical motor output map and their corresponding Stimulation Efficacy (SE). SE was the greatest value in the 80th percentile of the MEPs divided by the maximum stimulation intensity.

### Data Analysis

For statistical analysis, data were tested for normality using the Kolmogorov-Smirnov test. To identify significant time effects on clinical scores we performed a Friedman test. Next, we conducted a post-hoc analysis using 2-tailed Mann-Whitney *U* tests to compare improvements between groups at week 3 and week 12 follow-up. Within-subject analysis of recovery was assessed using standard clinical scales (Table 1). We reversed the polarity of Hamilton, VAS and Ashworth scales so that positive changes on all scales would express recovery.

In order to validate the RGS Adaptive Difficulty Controller, automatic performance ratios and difficulty parameters assigned by RGS to the paretic and non-paretic limb were compared (Wilcoxon signed-rank test). Next, to explicitly study progress in performance, we averaged values for each difficulty parameter per session and performed a within-subjects time-series analysis of the means (Friedman test).

Data of hand position and joint rotation collected during performance in AEMF were filtered using a second order Butter-worth low- pass filter (cut-off at 6 Hz) reducing noise. In order to assess the participant’s motor function within AEMF, we calculated three performance descriptors for each extremity: (1) the work area was defined as the dorsal surface area of the movement space, while (2) finger flexion, and (3) extension were defined as the maximal and minimal metacarpal angles respectively, averaged across all fingers.

We tested AEMF sensitivity by examining between-limbs differences in descriptor values (ie, covered area, finger flexion and finger extension) for each subject (Wilcoxon signed-rank test). Next, in order to explore AEMF test-retest stability and sensitivity to capture improvement, we analyzed changes in descriptor values across sessions (Friedman test). In addition, we studied the relation between standardized clinical scores and AEMF measurements of motor function by computing a Spearman correlation coefficient for each descriptor and clinical scale at the corresponding evaluation period.

Finally, we compared the Stimulation Efficacy (SE) and the centroid location of the cortical motor areas representing APB and ECR in M1, for the pathological and non-pathological hemispheres (Wilcoxon sign-sum test). In order to extract training effects, we performed a within-subject analysis of the Stimulation Efficacy and the centroid location of the cortical maps in M1 before and after treatment (Wilcoxon sign-sum test). We used a Spearman test to study the correlations between NBS outcome measures and improvements in clinical scales.

Two-sided significance level for all statistical tests was defined as alpha=0.05.Data processing and statistical analysis were performed using Matlab 2013a (MathWorks, Inc.). Due to limited statistical power, we did not correct for multiple comparisons.

**Table 1:** Participants’ demographics and scores from clinical scales at baseline.

Demographics	RGS (n=17)	Control (n=18)	*P* value
Gender (female), n (%)	9 (53)	12 (67)	.59^a^
Age, mean (SD)	65.05 (10.33)	61.75 (12.94)	.44^b^
Affected side (left), n (%)	11 (65)	9 (50)	.58^a^
Type (hemorrhagic), n (%)	6 (33)	6 (33)	.81^a^
Oxford class (LAC^c^/PAC^d^/TAC^e^)	4/3/4	6/2/4	.65^a^
Days after stroke, mean (SD)	1073.43 (767.74)	798.06 (421.80)	.64^b^
MMSE [16], mean (SD)	28.24 (2.33)	28.22 (2.34)	.08^b^
Hamilton [17], mean (SD)	3.71 (3.35)	4.56 (3.24)	.40^b^
Grip force, mean (SD)	6.15 (5.04)	5.94 (5.85)	.69^b^
MRC^f^ proximal [19], mean (SD)	3.47 (0.51)	3.39 (0.61)	.76^b^
MRC distal [19], mean (SD)	2.82 (1.19)	3.17 (0.99)	.44^b^
FMA [20], mean (SD)	42.94 (14.37)	43.44 (13.48)	.89^b^
CAHAI^g^ [21], mean (SD)	52.82 (23.10)	53.50 (22.51)	.95^b^
Barthel [22], mean (SD)	89.53 (9.43)	84.72 (14.19)	.48^b^
Ashworth proximal [23], mean (SD)	1.24 (1.25)	1.22 (1.31)	.97^b^
Ashworth distal [23], mean (SD)	1.47 (1.51)	1.00 (1.41)	.42^b^
VAS^h^ shoulder [16], mean (SD)	1.59 (2.76)	2.61 (2.64)	.13^b^

^a^Chi-square test.

^b^Wilcoxon rank-sum test.

^c^LAC: Lacunar stroke.

^d^PAC: Partial anterior circulation stroke.

^e^TAC: Total anterior circulation stroke.

^f^MRC: Medical Research Council.

^g^CAHAI: Chedoke Arm and Hand Activity Inventory (version CAHAI-13).

^h^VAS: Visual Analog Scale.

## Results

### Benefits of At-Home VR-Based Training on Motor Recovery

In order to assess the impact of the RGS treatment, we conducted a repeated measures analysis of the functional recovery captured through standardized clinical scales. Analysis of participants’ demographics revealed no significant differences between groups at baseline (Table 1). Comparing the change between baseline and week 3 in clinical scores we detected a significant difference on the CAHAI scale (Table 2). The RGS group showed significant improvements in CAHAI as compared to the control group (*P*=.05, Wilcoxon signed-rank test, Table 2). A post-hoc power analysis was conducted to determine the power of this statistical comparison for the sample size n=17. A medium effect size, d=0.48, at alpha=0.05 reached a low power level (Beta=0.4). A within-subjects analysis on the RGS group revealed an average improvement of 1.53 (SD 2.4) points on the CAHAI scale (*P*=.03, Wilcoxon signed-rank test); however, these effects did not persist at the week 12 follow-up evaluation. At follow-up we observed a significant difference between groups in improvement on the Ashworth scale only for distal muscle groups (*P*=.03, power=0.6, Wilcoxon signed-rank test), however, this difference did not reach statistical significance after Bonferroni correction.

### Progress of Performance in VR

Participants in the RGS group completed a variable total number of Hit (37.1, SD 18.4), Grasp (35.1, SD 17.0) and Place (34.2, SD 16.8) subtasks along the 3 weeks of treatment. All patients participating in the study were able to put the gloves on with assistance, and autonomously set-up and use the system until finishing the game. In order to assess whether the adaptive difficulty controller effectively provided customized training intensities that matched the participants’ capabilities, we explored inter-limb differences in mean performance ratios during training. Differences in performance showed a trend toward significance in Grasp and Place subtasks (*P*<.06, Wilcoxon). Notice that in order to provide an optimal training challenge for the user, the RGS system dynamically adjusted the difficulty parameters for each arm, mean performance ratios were maintained around 0.7 for each limb, across all tasks and sessions. Therefore these differences in performance between limbs may uncover existing floor effects in the difficulty adaptation algorithm for those participants unable to achieve complete power grasp movements [19]. In line with these findings, a within-subject analysis revealed a significant increase in the range and size difficulty coefficients assigned to the paretic limb during the Grasp and Place task across sessions (*P*<.05, Friedman). Similar improvements were observed for the non-paretic limb during the Hit and the Grasp subtasks.

### Automated Evaluation of Motor Function

**Figure 3:**
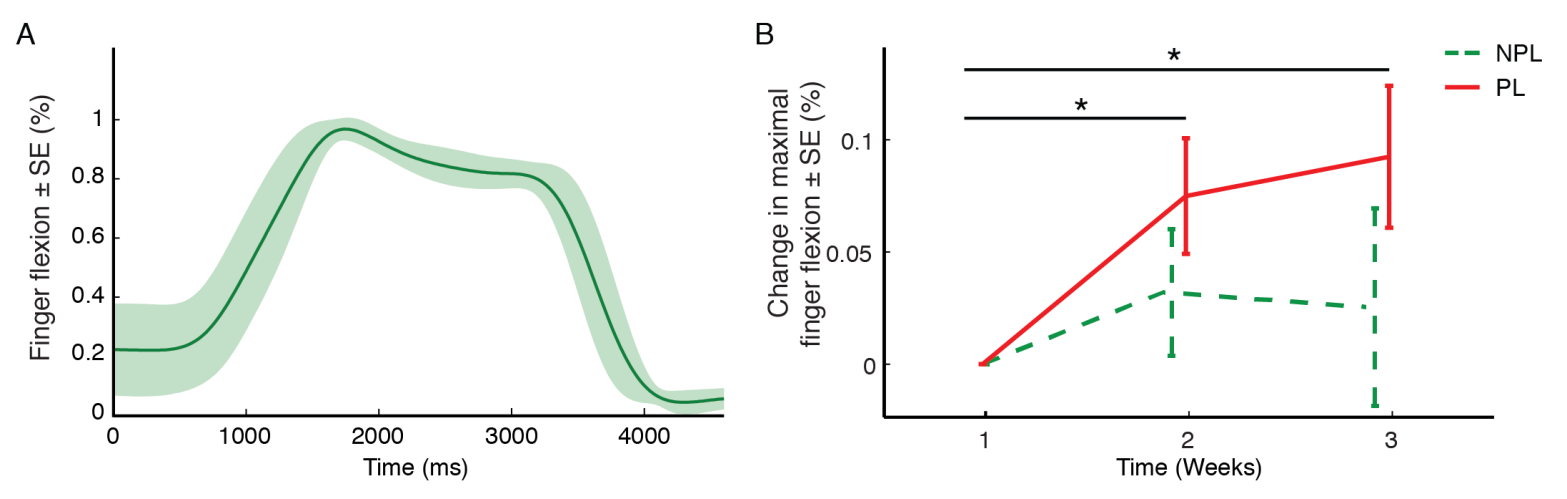
A: AEMF captures an improvement in finger flexion during treatment. Averaged movement profile of fingers excursion performed by one subject during one of the sessions. Units of finger flexion are expressed as a ratio of complete flexion. B: Mean changes in maximal finger flexion for all subjects in the RGS group across the three weeks of intervention, for both non-paretic (NPL) and paretic limbs (PL).

In order to study the RGS AEMF sensitivity, we compared measurements for the paretic and non-paretic limb. In addition, we explored the test-retest stability of these parameters. We observed that estimates of working area and maximal finger extension performed by the paretic limb in AEMF were significantly lower when compared to the non-paretic limb (*P*<.01, Wilcoxon). Within-subjects analysis showed no effect of time in the work area for the non-paretic (*P*=.06, Friedman), and a significant effect for the paretic limb (*P*=.03, Friedman). Post-hoc analysis revealed that these gains occurred during week 3 (*P*<.01, Wilcoxon). We also found a significant effect of time on maximum finger flexion for the paretic limb (*P*=.006, Friedman), which occurred at week 2 and 3 (*P*<.01, Wilcoxon, Figure 3). In order to validate AEMF, we correlated its measurements with assessments from standard clinical scales (Table 2). We used AEMF-derived improvement descriptors to fit scores from the CAHAI scale. An optimal fit was achieved by the sum of maximal finger flexion and extension (R-squared=.602, *P*<.001).

**Table 2:** Effects of RGS treatment versus control on clinical scales within and between groups for the post treatment assessment at week 3 and the long-term follow up at week 12.

Assessment	RGS (n=17)			Control (n=18)				Between Groups		Effect size
	Improvement, mean (SD)		*P*	Improvement, mean (SD)		*P*		*P*		Cohen *d*
End (Week 3)										
UE-FM^a^	0.35 (1.62)		.43	1.22 (3.84)		.15		.33		−0.30
CAHAI^b^	1.53 (2.4)		.01	−0.67 (6.01)		.90		.05		0.48
Barthel	0.00 (1.87)		>.99	1.00 (2.87)		.25		.44		−0.41
MRCp^c^	0.06 (0.24)		>.99	0.11 (0.32)		.50		.61		−0.17
MRCd^d^	0.06 (0.43)		>.99	0.11 (0.47)		.63		.74		−0.12
Asp^e^	0.00 (0.35)		>.99	0.06 (0.24)		>.99		.32		0.40
Asd^f^	0.12 (0.33)		.50	0.00 (0.34)		>.99		.32		0.36
Grip force	0.41 (1.78)		.89	0.38 (2.65)		.47		.57		0.01
Hamilton	0.88 (2.45)		.16	0.67 (1.57)		.13		.66		0.10
VAS-S^g^	0.41 (1.81)		.05	−0.28 (1.90)		.69		.63		0.37
						
Follow-up (Week 12)										
UE-FM	−0.18 (3.50)		.82	1.39 (3.63)		.11		.21		0.34
CAHAI	−0.06 (6.51)		.74	0.44 (5.46)		.67		.61		−0.08
Barthel	−3.30 (8.09)		.29	−0.11 (3.98)		.92		.74		−0.50
MRCp	−0.12 (0.78)		>.99	0.28 (0.46)		.06		.06		−0.62
MRCd	0.29 (0.77)		.25	0.17 (0.62)		45		.98		−0.17
Asp	0.06 (0.65)		>.99	0.00 (0.34)		>.99		>.99		−0.12
Asd^f^	0.29 (0.59)		.13	0.00 (0.00)		>.99		.03		0.70
Grip force	0.21 (1.45)		.73	0.23 (3.02)		.92		.93		−0.01
Hamilton	0.35 (2.34)		.70	1.11 (3.53)		.42		.93		−0.25
VAS-S	0.12 (2.06)		.92	0.78 (3.08)		.38		.27		−0.25
						
				

^a^UE-FM: The upper extremity Fugl-Meyer Assessment.

^b^CAHAI: Chedoke Arm and Hand Activity Inventory (version CAHAI-13).

^c^MRCp: Medical Research Council for proximal muscles.

^d^MRCd: Medical Research Council for distal muscles.

^e^Asp: Ashworth Scale for proximal muscles.

^f^Asd: Ashworth Scale for distal muscles.

^g^VAS-S: Visual Analog Scale for Shoulder Pain.

### RGS Induced Changes in the Corticospinal System

**Figure 4:**
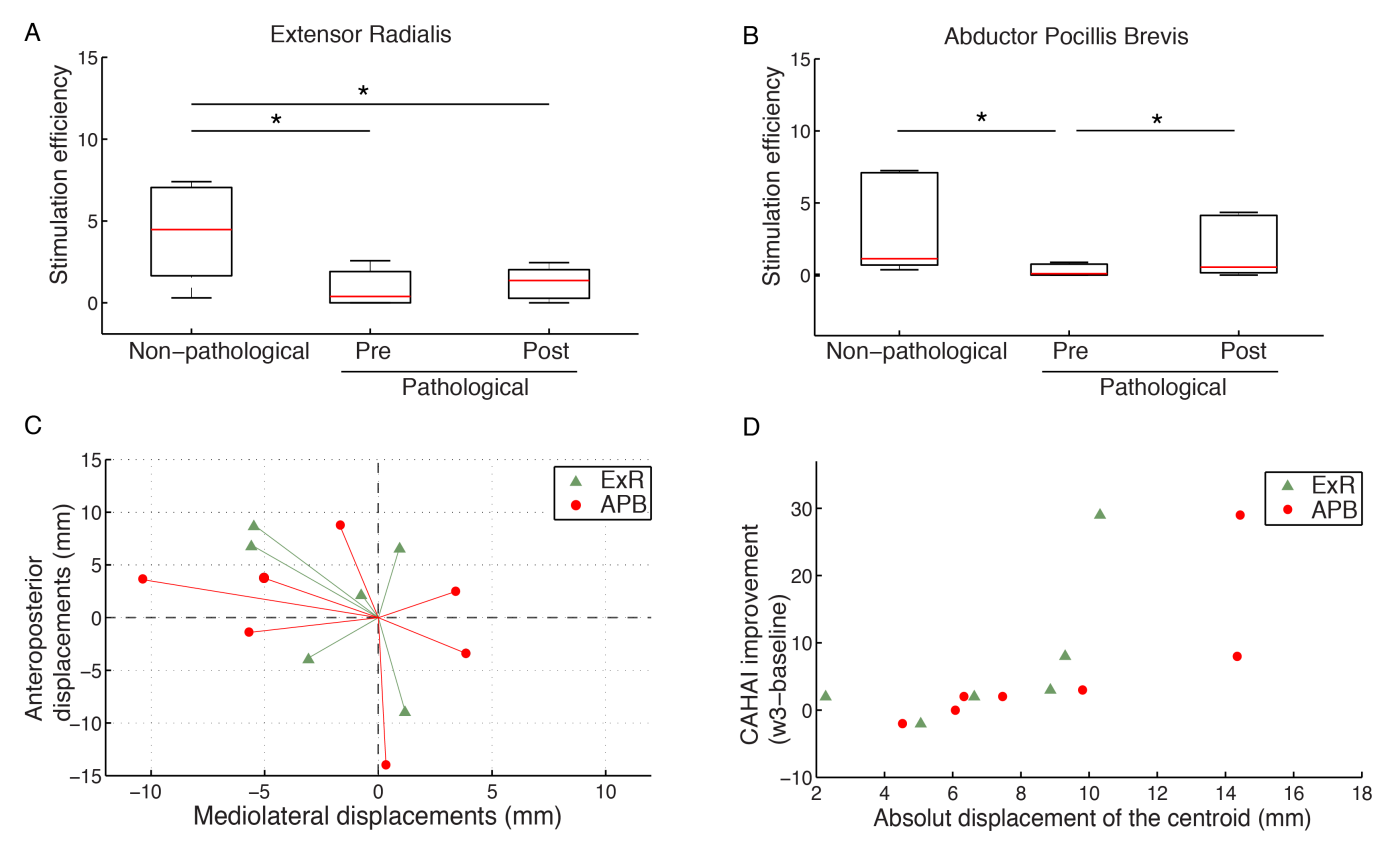
Effects of domiciliary rehabilitation therapy on corticospinal efficacy. (A) Change in mean Stimulation Efficacy for extensor-carpi radialis (ECR) in the damaged hemisphere (pathological) and the intact hemisphere (non-pathological). (B) Change in mean Stimulation Efficacy for abductor pollicis brevis (APB). (C) Centroid displacements after therapy along anterioposterior and mediolateral axis. (D) Correlation of absolute centroid displacements after therapy with improvement in CAHAI score after therapy.

In order to detect training-induced changes in the corticospinal system, we first characterized cortical regions in the primary motor area of the pathological and non-pathological hemispheres representing abductor pollicis brevis (APB) and extensor-carpi radialis (ECR) muscles. At baseline, the Stimulation Efficacy (SE) was significantly higher for the non-pathological hemisphere when compared to the pathological one (*P*<.01, Wilcoxon) (Figure 4). We observed that the centroid of the cortical area that produced MEPs was different between hemispheres along the mediolateral, and the anteroposterior axis (*P*<.05, Wilcoxon). In the non-pathological hemisphere, the cortical substrate representing the ECR was significantly larger than the area corresponding to the APB muscle (*P*<.05, Wilcoxon). Interestingly, this difference was not present in the pathological hemisphere.

SE increased significantly within subject after treatment in the pathological hemisphere (3.6, SD 8.60; *P*<.01, Wilcoxon). This change was significant only for the RGS group (4.17, SD 9.86; *P*<.01, Wilcoxon; *d*=.6) and the APB muscle (5.21, SD 10.98; *P*=.05, Wilcoxon; *d*=.66) [29].

We observed a centroid displacement in the pathological hemisphere, which occurred after treatment both for the APB and the ECR muscle (Figure 4). Since changes in cortical organization may indicate actual motor gains, we correlated post-treatment changes in SEs and centroid displacements with improvements at week 3 that were captured by the clinical scales [30]. Changes in SEs for the APB muscle strongly correlated with improvements in UE-FM (*r*_s_=.86, *P*<.01) (Figure 4), CAHAI (*r*_s_=.92, *P*<.01), and Barthel (*r*_s_=.68, *P*<.05), while the same effect was not present in the ECR muscle (*r*_s_<.61, *P*>.14). In addition, centroid displacements measured after intervention for the APB muscle were strongly correlated with UE-FM (*r*_s_=.87, *P*<.05), CAHAI (*r*_s_=.99, *P*<.01), and Barthel (*r*_s_=.81, *P*<.05). Centroid displacements for the ECR muscle also showed strong correlations with UE-FM (*r*_s_=.99, *P*<.01), and CAHAI (*r*_s_=.89, *P*<.05) clinical scales. Changes in the area of the cortical regions associated with each of the two muscles did not show any significant correlation with the improvements in clinical scales.

## Discussion

### Principal Findings

We have studied the effectiveness of the RGS VR-based system for home-based motor rehabilitation of the upper extremities after stroke by conducting a controlled, longitudinal clinical trial assessing both functional and structural impact and comparing it to an OT task. We have shown that, at the chronic stage post-stroke, the remote delivery of customized self-managed motor training in VR environments may effectively induce motor gains and neuroplastic changes. Comparisons between groups suggest a superiority of VR compared with OT in domiciliary setups, however, this difference does not reach clinical impact. Our results highlight the potential of automated rehabilitation technologies for domiciliary neurorehabilitation, which so far has been an issue of some contention [31].

First, we validated the RGS Adaptive Difficulty Controller, which automatically provides for a limb specific customization of practice difficulty and intensity, and a progress-monitoring tool. We observed lower success rates during the execution of those subtasks involving distal movements (ie, Grasp and Place). Lateralized customization of task difficulty allowed for the maintenance of optimal performance levels for each limb across sessions. Within-subject analysis of the evolution of the difficulty parameters assigned during training revealed paretic limb specific functional improvements during a reaching and grasping task. These observations may indicate functional gains of distal function (ie, increased control in fingers flexion and extension). Data collected by the Automated Evaluation of Motor Function further confirmed these findings, revealing significant improvements for the paretic limb, during week 2 and 3, in finger flexion. Interestingly, we also found an improvement in range of movement both for the paretic and non-paretic limb, probably indicating a generalization of new cognitive and compensatory strategies. Notice that subjects included in this study were in the chronic phase of stroke (mean time post stroke 65.05 months, SD 10.3), a period in which motor improvements are supposed to have plateaued and limited non-compensatory functional gains can still be induced through further physical or OT [32]. We show that the RGS group displayed significant gains on the CAHAI scale as compared to control. However, these changes did not reach the minimal detectable change (MDC=6.3 points) and we observed no retention of the improvements at follow-up, suggesting that achievement and retention of clinically relevant improvements at the chronic stage post-stroke may depend on longer intervention periods [30]. We did not observe any significant changes in the UE-FM scale, in any of the groups, perhaps due to the lack of responsiveness of this scale at the chronic stage post-stroke [33]. An alternative explanation for the lack of effect in the UE-FM scale is that these improvements may be fundamentally related to compensatory changes at the Body Functions and Structure and Activity levels [34].

Results from the NBS protocol supported these findings by displaying an enhanced corticospinal excitability after treatment only for the more distal muscle (ABP) associated with hand function. In addition, we observed centroid displacements of the cortical map for both the ABP and the ECR. This confirms earlier reports that enhanced corticospinal excitability and cortical map centroid displacements strongly correlate with functional gains detected by standardized clinical scales, such as Fugl-Meyer, CAHAI, and Barthel scales [30,32,35-37]. Previous research suggests that an imaging measure of corticospinal tract (CST) injury in the acute phase can predict motor outcome at 3 months [38]. Our results show that NBS-derived measures of corticospinal connectivity may be also relevant biomarkers for identifying chronic stroke survivors who have the potential to respond to a particular rehabilitative therapy and may be predictive of patient prognosis. Overall, these plastic changes may be use-dependent; an increase in the use of the paretic limb during the intervention period may have unmasked preexisting excitatory connections or even enhanced the efficacy of existing neuronal networks. Thus, RGS-induced cortical changes could be related to a greater activation in the ipsilesional hemisphere, as has been proposed by previous studies [39,40].

### Limitations

Taking a global perspective on these results, we observe that task difficulty descriptors, AEMF measurements, and NBS, converged, suggesting that distal functional improvements were induced through RGS based training and were significantly larger for those participants in the RGS group when compared with the control group. The reason why we may not have observed improvements in proximal muscle groups and other clinical scales may be related to the stringent inclusion criteria of the study, which excluded all subjects showing severe hemiparesis at baseline (Proximal Medical Research Council, MRC>2). It is widely known that the corticospinal system is organized following a proximal to distal gradient to the cervical spinal cord, where motoneurons of the distal muscle groups receive most input projections [41]. Due to this hierarchical organization, the severity of hemiparesis is often greatest in the distal muscles and least in the proximal muscles of the upper extremity [42]. Interestingly, this disparity may only appear at the chronic stage [18]. Consistent with these observations, participants showed a greater muscle weakness at baseline for distal than for proximal muscle groups (Table 1), which may be associated with a distal to proximal recovery process at this later stage post-stroke [43]. The specific factors involved in causing the observed RGS-derived improvements in distal function as compared to OT are not fully explained by our results. For instance, training in these two conditions differed in some aspects. On the one hand, RGS explicitly prevented the execution of compensatory body movements by capturing only joint flexion and extension and filtering out the participant’s trunk movements [44]. In contrast, participants in the control group, who followed a domiciliary OT protocol, without any supervision, may not have reached sufficient training intensity or may have reinforced the execution of functional compensatory movements (eg, overusing the non-paretic limb or performing trunk displacements) [45]. On the other hand, participants assigned to the RGS group repeatedly performed goal-oriented visuomotor transformations in order to control the virtual analogue of their paretic and non-paretic limbs, which may induce increased neural activity in cortical motor areas [40,46]. Indeed, we have shown that in healthy controls exposure to the RGS scenario leads to significantly enhance activity in premotor areas [47]. The OT group, however, was not exposed to such transformations, indeed subjects in this group performed repetitive visuomotor tasks in the real world only, where visual exposure to motor movements performed with the paretic limb may not be critical for successful performance. Although these are factors that could be better controlled in OT, our objective was to achieve a fair comparison between RGS virtual reality based and standard domiciliary OT and to understand their relative impact. In addition to motor gains, we observed a reduction in shoulder pain in the VR group, captured by the VAS scale. The reason for this effect may be that the VR group did not have to perform repetitive movements at the shoulder joint, unlike the control group. This difference could also explain the trend in an increase in muscle strength for the proximal musculature in the control group.

### Conclusions

In this randomized controlled study, we explored the effects of a VR-based system for domiciliary rehabilitation on functional recovery and cortical reorganization. Our results suggest that at-home VR-based rehabilitation promotes functional motor gains, enhances corticospinal excitability, and induces cortical reorganization at the chronic stage post- stroke. The observation of strong correlations between increased motor evoked potentials after treatment and functional gains in CAHAI suggests that exposure to VR-based goal-oriented motor training may have enhanced the organization of corticospinal pathways, facilitating distal motor control. The displacement of the centroid of cortical maps after training may also indicate related cortical reorganization at the chronic stage post-stroke supporting the idea that recovery can be induced at any stage post stroke albeit to varying degrees.

## Appendix

Multimedia Appendix 1 CONSORT eHealth form.

## Abbreviations

AEMF: automated evaluation of motor function
APB: abductor pollicis brevis
ASd: Ashworth scale for distal upper limb
ASp: Ashworth scale for proximal upper limb
BI: barthel index
CAHAI: chedoke arm and hand activity inventory
ECR: extensor-carpi radialis
MMSE: mini-mental state evaluation
MRC: medical research council scale
NBS: navigated brain stimulation
OT: occupational therapy
RGS: rehabilitation gaming system
SE: simulation efficacy
UE-FM: the upper extremity Fugl-Meyer assessment
VAS: visual analog scale
VR: virtual reality
